# Relationship between Subjective Oral Health Status and Lifestyle in Elderly People: A Cross-Sectional Study in Japan

**DOI:** 10.1155/2013/687139

**Published:** 2013-05-13

**Authors:** Masami Yoshioka, Daisuke Hinode, Masaaki Yokoyama, Aiichiro Fujiwara, Yasuhiko Sakaida, Kenji Toyoshima

**Affiliations:** ^1^Department of Oral Health Science and Social Welfare, Institute of Health Biosciences, The University of Tokushima Graduate School, Tokushima 770-8504, Japan; ^2^Department of Hygiene and Oral Health Science, Institute of Health Biosciences, The University of Tokushima Graduate School, Tokushima 770-8504, Japan; ^3^Medical Corporation Smile-kai Yoshida Dental Clinic, Minoh City, Osaka 562-0036, Japan; ^4^Kagawa Dental Association, Takamatsu City, Kagawa 760-020, Japan

## Abstract

*Objective*. The aim of this study was to assess the relationship between subjective oral health status and lifestyle in elderly people living in Japan. *Methods*. Subjects were 5383 inhabitants of the Kagawa Prefecture, Japan, at the age of 75–100. Records of the number of remaining teeth and the data from self-reported questionnaire were analyzed statistically. *Results*. Remaining teeth were significantly correlated to “no current smoking,” while not related to other lifestyle. On the other hand, “subjective masticatory ability” defined as a condition allowing chewing all foods well was related to favorable lifestyles. “Subjective masticatory ability” was also related to “not feeling stress,” “no deviated food habit” as well as to other good oral health conditions. A logistic regression analysis for “remaining teeth more than 20” revealed a significant relationship between “no current smoking” (OR = 1.582) and “no alcohol drinking” (OR = 0.851). Regarding “subjective masticatory ability,” all favorable lifestyles analyzed in this study were found to be significant positive factors. *Conclusions*. “Subjective masticatory ability” seems to be strongly associated with favorable lifestyles. Therefore, it can be suggested that “subjective masticatory ability” might be a good landmark for quality of life of elderly people in addition to the number of remaining teeth.

## 1. Introduction

From the time the “8020 Movement” was proposed by Japan Dental Association and approved by the Japanese Ministry of Health and Welfare in 1989 in Japan; the concept that maintaining more than 20 teeth at 80 years old leads to healthy and long life has been recognized throughout the country. Several studies conducted with “8020 achievers” reported that “8020 achievers” are better than “8020 nonachievers” for not only masticatory ability but also general physical conditions, including bone mineral density (BMI), grip strength, and the duration of balance test [1]. The food intake survey demonstrated that the “8020 achievers” ate a larger variety of food, although these foods were with less kilocalories and lower levels of carbohydrate [2].

Several epidemiological studies showed that the number of remaining teeth is closely related with smoking habit [3, 4] and with education level [4, 5].

A study investigating the masticatory ability of elderly people reported that “objective masticatory ability” was related significantly to “tooth loss less than 10” and “subjective evaluation that I can chew all foods well” [6]. This study also demonstrated that the masticatory ability of elderly people could affect the physical activity and maintenance of the social contacts, which are both related to quality of life (QOL) [6].

Although many studies indicated that maintaining more than 20 teeth provided a general health condition in elderly people, its relationship between oral health conditions and lifestyle except for smoking habit is still unclear.

The aim of this study was to clarify the relationship between lifestyle and “maintaining more than 20 teeth,” and to compare with the “subjective masticatory ability” which is defined as the capacity to chew all foods well.

## 2. Materials and Methods

This study was performed on 5383 elderly people (male; 2347, female; 3036, 75–100 years old, mean age; 80.2 ± 4.3) who participated in “the survey for present teeth number and oral health of elderly people” in Kagawa Prefecture in 2009. The data regarding the number of remaining teeth, collected by dentists belonging to Kagawa Dental Association, and the questionnaire concerning lifestyle and self-perceived oral health conditions was analyzed statistically. Remaining tooth was defined as a functional tooth for mastication.

The recategorized questionnaires are shown in Table 1. A bivariate analysis using the chi-squared test was conducted to determine associations in the distribution between study variables and “remaining teeth more than 20” or “subjective masticatory ability.” Logistic regression analysis for “remaining teeth more than 20” or “subjective masticatory ability” as the outcome variable was utilized to enumerate the odds ratio (OR) and 95% confidence interval (CI) relative to the reference group. All computations were conducted with SPSS 15.0 J software. The level of significance was considered as *P* < 0.05. The Ethics Committee of Tokushima University Hospital approved this study (the number of the protocol approval: 1121).

## 3. Results

### 3.1. Relationship between “Remaining Teeth More Than 20” and “Subjective Masticatory Ability”

The chi-squared test demonstrated a significant relationship between “remaining teeth more than 20” and “subjective masticatory ability” (*P* < 0.001) (Table 2). Twenty-nine percent of total subjects had “remaining teeth less than 20” but answered that “I can eat all food well.”

### 3.2. Relationship between “Remaining Teeth More Than 20” and the Various Items of Questionnaires

Table 3 shows the relationship between “remaining teeth more than 20” and study variables analyzed in males and females separately. A significant difference of “drinking alcohol” was observed in males, whereas it was not observed in females. No significant relationship was detected between “remaining teeth more than 20” and “regular life rhythm,” “regular sleep,” and “regular physical activity.” Only “no current smoking” was significantly related to “remaining teeth more than 20” (*P* < 0.01).

On the other hand, a significant association was observed with oral hygiene habit and eating behavior such as “observing teeth and/or gum using mirror,” “using an interdental brush and/or dental floss,” “spending enough time for tooth brushing,” and “no deviated food habit” by the chi-squared test (*P* < 0.01). Self-perceived oral health condition such as “not choked”, “no dry mouth,” and “chewing more than 30 times” was not related. The relation with “no feeling stress” was significant in females, but not significant in males.

### 3.3. Relationship between “Subjective Masticatory Ability” and the Various Items of Questionnaires

Table 4 shows the relationship between “subjective masticatory ability” and study variables analyzed in males and females separately. It was found that “subjective masticatory ability” was related significantly to “regular life rhythm,” “regular sleep,” “regular physical activity,” and “no current smoking” (*P* < 0.01). On the contrary, the association with “no drinking alcohol” was not significant neither in male nor female.

The chi-squared test showed a significant relation between “subjective masticatory ability” and all variables analyzed concerning “stress,” “oral hygiene habit,” “self-perceived oral health condition,” and “eating behavior.” These significant relations were demonstrated both in males and females.

### 3.4. Binominal Logistic Regression Analysis with “Remaining Teeth More Than 20” as the Dependent Variable Concerning “Lifestyle”

As shown in Table 5, variables significantly associated with “remaining teeth more than 20” were “no current smoking” (OR = **1.582**, 95%CI: **1.240–2.020**) and “no drinking alcohol” (OR = **0.851**, 95%CI: **0.749–0.966**).

### 3.5. Binominal Logistic Regression Analysis with “Subjective Masticatory Ability” as the Dependent Variable Concerning “Lifestyle”

Odds ratios (ORs) and their 95% confidence interval (95% CI) of various variables are shown in Table 6. Statistically significant associations were found between “subjective masticatory ability” and all variables concerning “lifestyle” except for “no drinking alcohol”.

## 4. Discussion

The National Health Promotion Movement in the 21st Century (Healthy Japan 21) [7] identified lifestyle-related diseases as targets and provided goals for these targets, aiming their implementation by 2010. These activities are intended to cover nine specific areas including dental health. Result from this study revealed that “subjective masticatory ability” was clearly related to “favorable lifestyle” such as “regular life rhythm,” “regular sleep,” and “regular physical activity” in contrast with “remaining teeth more than 20.” We found that the persons who can chew all foods well with less than 20 present teeth represented 29.4% of all subjects. Actually, it was more than the person who can chew all foods well with more than 20 present teeth (1392 persons versus 1581 persons). In fact, “8020 Movement” originated from the consensus that they can chew all foods well if they have more than 20 teeth. Data from this study might suggest that the goal which we should aim at is “maintaining to chew all foods well” in addition to “maintaining teeth more than 20.”

In this study, we found that “subjective masticatory ability” was significantly related to “self-perceived good oral conditions” (i.e., “chewing more than 30 times before swallowing,” “not choked,” “no dry mouth”). In addition, a significant relationship with “no deviated food habit” was also found. It might be speculated that the ability to chew all foods well leads to balanced food intake, stimulation of saliva secretion followed by safety swallowing. In other words, enough masticatory ability might provide to elderly people some advantages for maintaining oral functions, balanced nutrition, and food intake, maintaining physical power and activity, preventing a stress derived from trouble when eating, and at last, improving quality of life.

The binominal logistic regression analysis with “remaining teeth more than 20” showed that “no drinking alcohol” was a significant negative factor. These findings are consistent with previous reports [8, 9]. Hanioka et al. demonstrated that “drinking alcohol” was negatively associated with “total tooth loss” in elderly women in Japan [8]. Nakayama and Mori reported a significant relationship between “remaining teeth fewer than 20” and “drinking alcohol” in elderly men [9] in Japan. On the other hand, it is also reported that “heavy drinking” was associated with tooth loss [10]. In this study, we could not distinguish the amount and/or intake frequency of alcohol. Since many “light drinker” might be categorized as “drinker” in this study, we cannot conclude that “no drinking alcohol” is favorable lifestyle.

Since this study was a cross-sectional investigation, we cannot conclude causality of each variable analyzed. It was confirmed that “current smoking” affected strongly the present teeth number, as previously reported. We must emphasize more to reduce tobacco use towards young smoker for maintaining teeth in future and for enjoying meal time without any trouble at old age.

In conclusion, it is suggested that subjective masticatory ability is associated with daily life habits. Therefore, general health care should be considered by those responsible for oral management to support the QOL of elderly people.

## 5. Conclusions

Our results suggest that subjective masticatory ability which enables to chew all foods well was significantly related to favorable oral health conditions. Besides, “subjective masticatory ability” was associated with daily living habit such as regular sleep and regular physical activity, while “remaining teeth more than 20” was not. It is suggested that “subjective masticatory ability” might be a good landmark for QOL of elderly people in addition to the number of remaining teeth.

## Figures and Tables

**Table 1:** Items of the recategorized questionnaires in this study.

Lifestyle	
(1) Do you live a regular life rhythm? (Yes, No)	
(2) Do you have a regular sleep? (Yes, No)	
(3) Do you perform regular physical activity? (Yes, No)	
(4) Do you have current tobacco habit? (No, Yes)	
(5) Do you drink alcohol? (No, Yes)	
Stress	
(6) Do you feel stressed? (No, Yes)	
Oral hygiene habit	
(7) Do you observe your teeth and/or gum using a mirror? (Yes, No)	
(8) Do you use an interdental brush and/or dental floss? (Yes, No)	
(9) Do you spend enough time for tooth brushing? (Yes, No)	
Subjective oral health symptom	
(10) Have you choked? (No, Yes)	
(11) Do you feel mouth dry? (No, Yes)	
Eating behavior	
(12) Do you have a deviated food habit? (No, Yes)	
(13) How many times do you chew before swallowing? (more than 30 times, fewer than 30 times	
Subjective masticatory ability	
(14) Can you chew all foods well? (Yes, No)	

**Table 2:** Distribution of numbers and percentages of subjects by “remaining teeth number” and “subjective masticatory ability.”

	Can you eat all foods well?		Total
	Yes	No	
Remaining teeth			
More than 20	1392 (25.9%)	502 (9.3%)	1894 (35.2%)
Less than 20	1581 (29.4%)	1908 (35.4%)	3489 (64.8%)

Total	2973 (55.3%)	2410 (44.7%)	5383 (100%)

**Table 3:** Distribution of subjects who have “remaining teeth more than 20 teeth” by study variables (Chi-squared test).

Variable	Male(*N* = 2347)			Female (*N* = 3036)		
	N*	%**	*P *	N*	%**	*P *
Regular life rhythm						
Yes	2174	35.1%	0.922	2815	35.4%	0.657
No	173	34.7%		221	33.9%	

Regular sleep						
Yes	1882	35.8%	0.132	2228	35.3%	0.979
No	465	32.0%		808	35.3%	

Regular physical activity						
Yes	1244	35.9%	0.371	1400	36.4%	0.264
No	1103	34.1%		1636	34.4%	

Current smoking						
No	2068	36.0%	0.005	2958	35.6%	<0.001
Yes	279	27.6%		78	24.4%	

Drinking alcohol						
No	1316	32.9%	0.014	2608	35.1%	0.521
Yes	1031	37.7%		428		

Feeling stress						
No	441	32.4%	0.204	490	30.8%	0.023
Yes	1906	35.6%		2546	36.2%	

Observation of teeth and/or gum						
Yes	989	40.2%	<0.001	1461	40.8%	<0.001
No	1358	31.2%		1575	30.2%	

Using interdental tools						
Yes	864	45.9%	<0.001	1284	48.6%	<0.001
No	1483	28.7%		1752	25.6%	

Enough time for tooth brushing						
Yes	1029	40.1%	<0.001	1334	40.0%	<0.001
No	1318	31.0%		1702	31.7%	

Being choked						
No	1760	35.3%	0.577	2280	36.0%	0.161
Yes	587	34.1%		756	33.2%	

Feeling mouth dry						
No	1522	35.9%	0.240	1776	36.5%	0.091
Yes	825	33.5%		1260	33.6%	

Deviated food habit						
No	1888	36.4%	0.005	2438	36.5%	0.004
Yes	459	29.4%		598	30.3%	

Chewing before swallowing						
More than 30 times	456	37.7%	0.178	584	35.6%	0.862
Fewer than 30 times	1891	34.4%		2452	35.2%	

*N: total number of subjects corresponding to each answer.

**%: the percentage of subjects who have “more than 20 teeth” corresponding to each answer.

**Table 4:** Distribution of subjects who can chew all foods well by study variables (Chi-squared test).

Variable	Male(*N* = 2347)			Female (*N* = 3036)		
	N*	%**	*P *	N*	%**	*P *
Regular life rhythm						
Yes	2174	57.7%	<0.001	2815	55.5%	<0.001
No	173	42.8%		221	37.1%	

Regular sleep						
Yes	1882	59.8%	<0.001	2228	57.8%	<0.001
No	465	43.7%		808	44.2%	

Regular physical activity						
Yes	1244	62.2%	<0.001	1400	62.0%	<0.001
No	1103	50.2%		1636	47.5%	

Current smoking						
No	2068	57.6%	0.007	2958	54.6%	0.002
Yes	279	49.1%		78	37.2%	

Drinking alcohol						
No	1316	56.6%	0.975	2608	54.7%	0.177
Yes	1031	56.5%		428	51.2%	

Feeling stress						
No	441	67.1%	<0.001	490	66.1%	<0.001
Yes	1906	54.1%		2546	51.9%	

Observation of teeth and/or gum						
Yes	989	59.0%	0.040	1461	57.8%	<0.001
No	1358	54.8%		1575	50.8%	

Using interdental tools						
Yes	864	64.2%	<0.001	1284	60.6%	<0.001
No	1483	52.1%		1752	49.5%	

Enough time for tooth brushing						
Yes	1029	61.5%	<0.001	1334	60.3%	<0.001
No	1318	52.7%		1702	49.4%	

Being choked						
No	1760	60.3%	<0.001	2280	58.2%	<0.001
Yes	587	45.5%		756	42.2%	

Feeling mouth dry						
No	1522	60.3%	<0.001	1776	60.5%	<0.001
Yes	825	49.7%		1260	45.2%	

Deviated food habit						
No	1888	59.5%	<0.001	2438	57.3%	<0.001
Yes	459	44.7%		598	41.5%	

Chewing before swallowing						
More than 30 times	456	61.2%	0.027	584	58.2%	0.029
Fewer than 30 times	1891	55.5%		2452	53.2%	

*N: total number of subjects corresponding to each answer.

**%: the percentage of subjects who can chew all foods well corresponding to each answer.

**Table 5:** Odds ratios and 95% confidence intervals for “remaining teeth more than 20” by binominal logistic regression analysis* (*n* = 5383).

Variable	Or	95% CI	*P*-value
Regular life rhythm			
Yes	0.985	0.784–1.237	0.896
No (ref.)			

Regular sleep			
Yes	1.053	0.916–1.211	0.468
No (ref.)			

Regular physical activity			
Yes	1.064	0.950–1.191	0.287
No (ref.)			

Current smoking			
No	**1.582**	**1.240–2.020**	**<0.001**
Yes (ref.)			

Drinking alcohol			
No	**0.851**	**0.749–0.966**	**0.013**
Yes (ref.)			

*Binominal logistic regression analysis was conducted using 5 variables concerning life-style as the dependent variable.

Results significant at the 5% level are indicated in bold.

ref.: reference category.

**Table 6:** Odds ratios and 95% confidence intervals for “subjective masticatory ability” by binominal logistic regression analysis* (*n* = 5383).

Variable	OR	95% CI	*P*-value
Regular life rhythm			
Yes	**1.436**	**1.150–1.793**	**0.001**
No (ref.)			

Regular sleep			
Yes	**1.617**	**1.414–1.849**	**<0.001**
No (ref.)			

Regular physical activity			
Yes	**1.641**	**1.470–1.833**	**<0.001**
No (ref.)			

Current smoking			
No	**1.420**	**1.137–1.773**	**0.002**
Yes (ref.)			

Drinking alcohol			
No	1.061	0.937–1.203	0.351
Yes (ref.)			

*Binominal logistic regression analysis was conducted using 5 variables concerning life-style as the dependent variable.

Results significant at the 5% level are indicated in bold.

ref.: reference category.
